# Gastroenterologist and primary care perspectives on a post-endoscopy discharge policy: impact on clinic wait times, provider satisfaction and provider workload

**DOI:** 10.1186/s12913-017-2819-6

**Published:** 2018-01-10

**Authors:** Daniel Selvig, Justin L. Sewell, Delphine S. Tuot, Lukejohn W. Day

**Affiliations:** 1 Department of Medicine, University of California, San Francisco, CA USA; 2 Division of Gastroenterology, Department of Medicine, Zuckerberg San Francisco General Hospital and Trauma Center, San Francisco, CA USA; 3 Division of Nephrology, Department of Medicine, Zuckerberg San Francisco General Hospital and Trauma Center, San Francisco, CA USA; 4 UCSF Center for Innovation in Access and Quality at Zuckerberg San Francisco General Hospital, San Francisco, CA USA; 5 San Francisco General Hospital and Trauma Center, 1001 Potrero Avenue, 3D-5, San Francisco, CA 94110 USA

**Keywords:** Quality of care, Provider satisfaction, Access to healthcare, Endoscopy, Gastroenterology, Wait times

## Abstract

**Background:**

To reduce unnecessary ambulatory gastroenterology (GI) visits and increase access to GI care, San Francisco Health Network gastroenterologists and primary care providers implemented guidelines in 2013 that discharged certain patients back to primary care after endoscopy with formal written recommendations. This study assesses the longer-term impact of this policy on GI clinic access, workflow, and provider satisfaction.

**Methods:**

An email-based survey assessed gastroenterologist and primary care provider (PCP) opinions about the discharge process. Administrative data and chart review were used to assess clinic access, intervention fidelity, and re-referral rates.

**Results:**

102/299 (34%) of PCPs and 5/7 (71%) of gastroenterologists responded to the survey. 74% of PCPs and 100% of gastroenterologists were satisfied or very satisfied with the discharge process. 80% of gastroenterologists believed the discharge process decreased their workload, while 53.5% of primary care providers believed it increased their workload. 6.7% of patients discharged to primary care in 2013 had re-referrals to GI. Wait time for the third-next-available new outpatient GI clinic appointment had previously decreased from 158 days (2012, pre-intervention) to 74 days (2013, post-intervention). In 2015, wait time was 19 days (*p* < 0.001 for 2012 vs. 2015).

**Conclusions:**

Primary care providers and gastroenterologists are satisfied with an intervention to discharge patients from gastroenterology to primary care after certain endoscopic procedures, although this conclusion is limited by a relatively low PCP survey response rate. Discharging appropriate patients using consensus criteria from the gastroenterology clinic was instrumental in sustainably reducing clinic wait times with low re-referral rates.

**Electronic supplementary material:**

The online version of this article (10.1186/s12913-017-2819-6) contains supplementary material, which is available to authorized users.

## Background

Utilization of ambulatory specialty services is increasing in the United States. In the decade from 1999 to 2009, the absolute number of visits that resulted in a referral more than doubled [1]. This problem has been especially challenging for patients served by safety net healthcare delivery systems. A survey of key safety net providers in California revealed that 85% of medical directors “often” or “almost always” have problems obtaining specialty care for uninsured patients, compared with 2% for patients with private insurance [2].

Discharging patients from specialist care to primary care when they can safely be managed in a primary care setting is one potential method of increasing access to specialist services. This strategy has been pursued in the United Kingdom (U.K.) where significant numbers of patients have been identified as suitable for discharge from specialty to primary care [3–5]. This strategy holds promise in the United States as well. An analysis of National Ambulatory Medical Care Survey data revealed that 46% of specialist visits in the United States were for follow-up visits of patients already known to the specialist [6]. It is possible that some of these patients may not require scheduled specialist follow-up. A survey of primary care providers (PCPs) and specialists from one academic medical center showed that for 16% of a sample of patients seen by specialists, both primary care and specialty providers agreed that the patient could be managed exclusively by the PCP [7].

The gastroenterology clinic at the Zuckerberg San Francisco General hospital (ZSFG) in partnership with its referring network of primary care providers (PCPs) developed an intervention to identify patients who could be safely discharged to primary care following endoscopy or colonoscopy [8]. In that study, wait time for third next available gastroenterology clinic appointment decreased by 53% and the ratio of new to follow-up visits increased in the 4 months following the implementation of the discharge policy. This initial study did not assess primary care or gastroenterologist satisfaction with the process after its implementation, and did not measure possible downstream effects of the intervention, such as a concentration of more complex patients evaluated in gastroenterology clinic or the frequency of re-referrals to gastroenterology for patients discharged to primary care. Understanding the long-term implications of a discharge policy between specialty and primary care could inform the feasibility of implementing a similar policy in other specialties.

The objective of the current study is to assess: 1) Satisfaction of PCPs and gastroenterologists with the discharge process, 2) Perceived impact of the discharge policy on primary care and gastroenterologist workload, 3) Frequency of re-referrals to gastroenterology following discharge to primary care after endoscopy, and 4) Ongoing fidelity and impact of the intervention on clinic access 2 years after implementation.

## Methods

### Setting

The San Francisco Health Network is the vertically integrated safety net healthcare delivery system for the uninsured and underinsured in San Francisco. It is comprised of ZSFG and two networks of primary care clinics. PCPs within this system can refer patients to the gastroenterology clinic at ZSFG. In 2013 the gastroenterology clinic implemented a policy by which certain patients could be discharged back to primary care following an upper endoscopy or colonoscopy, rather than having a scheduled visit with a gastroenterologist to follow up on results. The discharge criteria were developed using a modified Delphi process involving 130 PCPs and 7 gastroenterologists, which has previously been described in detail [8]. In brief, the process allowed PCPs and gastroenterologists to evaluate a number of clinical scenarios and achieve consensus for which patients could be safely discharged to primary care without planned gastroenterology follow-up. Discharge criteria are shown in an attached file (Additional file 1 – Discharge Criteria.pdf).

### Provider satisfaction survey

**Fig. 1:**
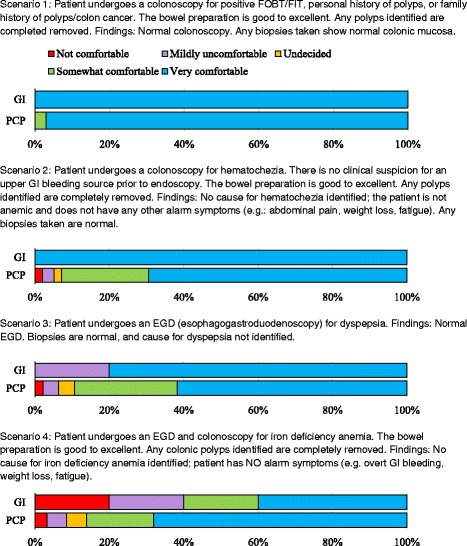
Respondent Comfort with Discharge Scenarios

Two hundred ninety nine PCPs and 7 gastroenterologists were invited via email to participate in an online survey (Additional files 2 and 3) distributed using REDCap, a secure web-based application designed to support data capture for research studies [9]. Surveys were completed in November 2015 (gastroenterologists) and March–April 2016 (PCPs). Following the initial email, one reminder email was sent to remind participants to take the survey. Respondents were not compensated and participation was anonymous. The survey asked providers to evaluate their comfort level with the existing gastroenterology discharge policy following endoscopy or colonoscopy for 4 clinical scenarios (Fig. 1). Participants were also asked their satisfaction with the discharge criteria and how it affected their workload. PCPs were asked about their satisfaction with written recommendations provide by gastroenterologists, and gastroenterologists were asked about the effects of the discharge policy on the complexity of their clinic patients.

### Long term intervention impact and fidelity -- chart review

A random week of January-April of 2013 and January-April of 2015 were selected for patient chart review to assess long-term fidelity and impact on gastroenterology clinic access. Patients who had undergone an ambulatory colonoscopy or endoscopy at ZSFG during these time periods were identified by searching the Provation endoscopy records program. The indication for procedure, adequacy of bowel preparation, and pathologic findings were reviewed to determine whether the patient met one of the criteria for discharge to primary care. The recommendations provided in the procedure note were reviewed to determine whether the patient was to be discharged to primary care. For patients who were discharged to primary care following endoscopy in 2013, patient records were reviewed (Lifetime Clinical Record medical records system) for the 2 years following the endoscopic procedure to determine whether the patient had a referral submitted to gastroenterology clinic related to the endoscopic procedure that had been performed. New referrals to gastroenterology for reasons unrelated to the original 2013 endoscopic procedure were not considered to be re-referrals. Wait times for third next available new patient appointment were collected from administrative data for a period of January-April of 2014 and 2015, and compared with data from 2012 and 2013 which has previously been published [8].

### Data analysis

Wait times in 2012 and 2015 were compared with t-tests. ANOVA tests were used to determine whether PCPs who had participated in the creation of the discharge criteria reported different levels of satisfaction with the discharge criteria compared to those who did not. For this analysis, the responses were treated as interval variables along a 5-point scale, with “very satisfied” equal to 5 and “very unsatisfied” equal to 1. *P* values of <0.05 were considered statistically significant. For qualitative data, comments were read and grouped into themes to identify major patterns in content.

### Ethical considerations

The study protocol including the survey, chart review, and administrative data analysis was submitted to the UCSF Committee for Human Research (CHR) and was found to meet criteria for a quality improvement study exempt from full review (Reference number 147400). Survey data were collected anonymously and were not linked to respondent email addresses or identifying information.

## Results

### Respondent characteristics

One hundred two out of 299 PCPs responded to the survey (response rate of 34%), and 86 answered every question in the survey. Five out of 7 gastroenterologists responded to the survey (response rate of 71%). Survey respondent characteristics are shown in Table 1. Of primary care respondents, 46.7% were attending physicians, 33.7% were residents or fellows, 17.4% were nurse practitioners, and 2.2% were physician assistants. PCPs most frequently reported working 1-2 clinical half-days per week (37.6%). A minority (23.5%) had participated in the initial modified Delphi process in 2012, although an additional 34.3% were not sure whether they had participated. Most PCPs (89.2%) had patients who had been discharged to primary care following endoscopy in accordance with the discharge guidelines. All gastroenterologists were attending physicians and had clinic 1-2 half days per week. 60% of gastroenterologists performed endoscopic procedures 1-2 half days per week.

**Table 1:** Survey Respondent Characteristics

	Primary care providers *N* = 102	Gastroenterologists *N* = 5
Respondent Characteristics	N (%)	N (%)
Physician (attending)	43 (46.7)	5 (100%)
Physician (resident or fellow)	31 (33.7)	
Nurse practitioner performing primary care	16 (17.4)	
Physician assistant primarily performing primary care	2 (2.2)	
Clinical half-days per week		
1–2 half days per week	35 (37.6)	4 (100.0)
3–4 half days per week	29 (31.2)	0 (0.0)
5–6 half days per week	19 (20.4)	0 (0.0)
At least 7 half days per week	10 (10.8)	0 (0.0)
Half days per week performing endoscopic procedures		
1–2		3 (60.0)
3–4		2 (40.0)
5–6		0 (0.0)
At least 7		0 (0.0)
Have had patients discharged by this process?		
Yes	83 (89.2)	5 (100.0)
No	5 (5.4)	
Not sure	5 (5.4)	

Numbers may not always sum to total n because of incomplete survey responses

### Provider perspectives on the post-endoscopy discharge policy

The majority of PCPs were satisfied with the post endoscopy discharge criteria with 74% reporting feeling either very satisfied or satisfied. Equally satisfied with the discharge criteria were gastroenterologists with 60% reporting feeling very satisfied and 40% feeling satisfied with the discharge process. Satisfaction with the discharge criteria did not vary significantly for PCPs based on whether they had participated in the original Delphi survey process. After converting responses to a 5-point scale where “very satisfied” is equal to 5 and “very unsatisfied” is equal to 1, those who had participated in the original Delphi survey process had a mean satisfaction 4.25/5 compared with those who had not participated (mean satisfaction 3.97/5) or were not sure (mean satisfaction 3.79/5) (*p* = 0.25). Nearly 54% of PCPs thought the discharge process either increased or slightly increased their workload for those patients discharged to primary care. Among gastroenterologists, 80% believed the discharge process lessened or slightly lessened their workload. Most PCPs were satisfied with post-discharge recommendations provided by gastroenterologists (34.9% were very satisfied and 46.5% were satisfied) and only 5.8% reporting feeling unsatisfied/very unsatisfied. All gastroenterologists believed that the average complexity of their clinic patients has increased since the implementation of the discharge process (Table 2). Comfort levels with specific discharge scenarios are shown in Fig. 1.

**Table 2:** Survey Responses

	Primary care providers *N* = 102	Gastroenterologists *N* = 5
Participant Responses	N (%)	N (%)
Satisfaction with discharge process		
Very satisfied	31 (35.2)	3 (60.0)
Satisfied	34 (38.6)	2 (40.0)
Neither satisfied nor unsatisfied	16 (18.2)	0 (0.0)
Unsatisfied	5 (5.7)	0 (0.0)
Very unsatisfied	2 (2.3)	0 (0.0)
Effect on Workload		
Lessens workload	5 (5.7)	3 (60.0)
Slightly lessens workload	5 (5.7)	1 (20.0)
No effect on workload	31 (35.2)	0 (0.0)
Slightly increases workload	40 (45.5)	1 (20.0)
Increases workload	7 (8.0)	0 (0.0)
Satisfaction with GI recommendations		
Very satisfied	30 (34.9)	–
Satisfied	40 (46.5)	–
Neither satisfied nor unsatisfied	11 (12.8)	–
Unsatisfied	3 (3.5)	–
Very unsatisfied	2 (2.3)	–
Effect on patient complexity		
More complex	–	4 (100.0)
Slightly more complex	–	0 (0.0)
No change	–	0 (0.0)
Slightly less complex	–	0 (0.0)
Less complex	–	0 (0.0)

PCPs were asked what additional comments they had about the gastroenterology clinic discharge process at the end of the survey, and were allowed to answer via free text. Responses included a variety of feedback, but two common themes emerged. Many PCPs commented on the process of finding the recommendations in the computer, for example: “The only reason why I’m not 100% satisfied with the process is that it’s often hard to find where the recommendations are located [in the electronic health record].” Secondly, several respondents commented on the importance of clear and thorough recommendations from the gastroenterologist: “In the given scenarios, I would be comfortable caring for patients without GI follow up as long as recommendations are comprehensive and explicitly stated.”

### Fidelity of discharge criteria

All endoscopic procedures during a random week in each month of January-April 2015 were reviewed to assess fidelity of the discharge criteria, totaling 111 upper endoscopies and 198 colonoscopies. Twelve of the 111 patients undergoing upper endoscopy met criteria for discharge, and 10 were actually discharged to primary care without scheduled gastroenterologist follow-up (83%). Of the 198 colonoscopies, 78 met criteria for discharge, and all 78 (100%) were discharged to primary care without scheduled gastroenterology follow-up.

### Impact of discharge criteria on wait times

**Fig. 2:**
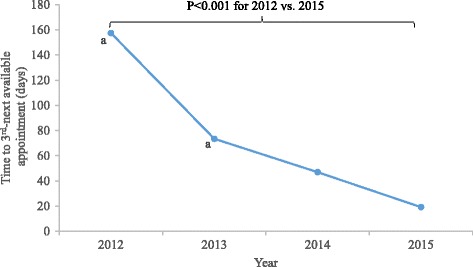
Clinic wait times. a) 2012 and 2013 data previously reported in Tuot et al. 2014 [8]. Wait times for 3rd-next available new patient appointment, for a sample period of January-April of each year. *P* < 0.001 for difference in wait time between 2012 and 2015

As previously reported, wait times for the third next available appointment decreased from 158 days in January-April 2012 to 74 days in January-April 2013 after implementation of discharge criteria [8]. Wait time to third next available appointment for January-April 2014 was 47 days, and for January-April 2015, wait time was 19 days (Fig. 2). The difference in wait time between 2012 (pre-intervention) and 2015 (2 years after implementation of the discharge policy) was statistically significant (*p* < 0.001).

### Re-referral rates back to gastroenterology by primary care

Chart review of randomly chosen weeks between January-April 2013 demonstrated that 1/13 (7.7%) patients discharged after upper endoscopy and 5/76 (6.6%) patients discharged after undergoing colonoscopy had re-referrals to gastroenterology within 2 years for reasons related to the original procedure. Of the 6 patients re-referred to gastroenterology, two re-referrals were questions regarding the appropriate colonoscopy follow-up interval and four re-referrals originated because no recommendations had been left in the chart by the gastroenterologist. All 6 of the re-referrals were handled electronically via an electronic consultation system rather than through in-person consultations.

## Discussion

In this study, we show that PCPs and gastroenterologists are generally satisfied with an intervention to discharge patients from gastroenterology clinic to primary care using consensus discharge criteria. These levels of satisfaction are despite a slight increase in perceived workload reported by PCPs, and a perceived increase in complexity of patients seen in gastroenterology clinic. PCP satisfaction was not significantly different for PCPs who had participated in the original Delphi process from which the discharge criteria were developed compared with those who had not. Furthermore, discharging appropriate patients using these consensus criteria was instrumental in sustainably reducing clinic wait times with low re-referral rates.

These results are consistent with previous research assessing primary care perspectives regarding the discharge of patients from specialty to primary care. A study using semi-structured interviews with primary care physicians in the UK revealed that many felt largely positive regarding the discharge of patients from regular specialty care follow-up to primary care, but expressed a desire for better communication, guidance on future management from the specialist, and quick access to specialty care if re-referral is needed [3]. Another study using focus groups of PCPs described facilitators and barriers of the transition from specialty diabetes care to primary care [10], finding that clear communication of a structured plan, ongoing access to specialist services, and continuing education of PCPs were major facilitators of successful transition. Satisfaction and opinions of primary care and specialist providers have not been previously reported for a post-procedural setting such as endoscopy; our research adds to this literature by showing an example of a discharge policy in a post-procedural setting that is generally well accepted by primary care physicians and gastroenterologists.

Comfort levels with the discharge criteria were generally similar to those found in the original Delphi survey process [8] with high levels of comfort reported for patients with abnormal fecal occult blood test or fecal immunochemistry (FOBT/FIT) testing but normal colonoscopy results as well as for patients with hematochezia and normal colonoscopy results (Fig. 1). Differences with the original Delphi survey process were noted for patients with dyspepsia and normal upper endoscopy results, for whom 20% of gastroenterologists were “mildly uncomfortable” discharging whereas 100% previously reported feeling very comfortable or somewhat comfortable discharging. Providers’ comfort discharging patients with iron deficiency anemia without alarm symptoms and a normal colonoscopy also differed from results of the prior Delphi survey; 60% of gastroenterologists in the current study were somewhat comfortable/very comfortable discharging these patients, whereas in the original study, only 30% of gastroenterologists were somewhat comfortable/very comfortable.

Data on the prevalence of different follow-up strategies after endoscopy are generally lacking, however there are some data regarding post-endoscopy follow-up methods in a safety net setting. In a survey of endoscopy centers at public hospitals in California, in-person follow-up appointments were the most commonly used method of communicating biopsy results (75% of centers) compared with letters to patients (12.5%), telephone calls to patients (25%) and being discharged back to primary care to follow up with the referring physician (50%) [11]. The gastroenterology clinic at ZSFG benefits from sharing an electronic medical record with many, though not all, of its referring PCPs. The ZSFG gastroenterology clinic also benefits from the use of an integrated electronic consultation and referral system [12], which facilitates re-referrals, if necessary. Practices without these advantages may have difficulty implementing a post-endoscopy discharge policy due to challenges in ensuring effective post-discharge communication.

Our study also addressed the crucial issue of provider workload. PCPs reported that the discharge process generally increased their workload, while gastroenterologists tended to report that it lessened their workload. If significant numbers of patients were discharged to primary care from multiple specialty types, primary care workload could potentially increase further. A recent study suggested that a typical PCP has 229 other physicians also caring for members of his/her patient panel with Medicare [13]. With ongoing changes to the primary care-specialty care interface, primary care practices will need adequate resources to provide and coordinate care, and primary care access and workload will need to be monitored. Team-based models of care such as the patient-centered medical home may be helpful to ensure high-quality care coordination for these patients seeing multiple providers.

Gastroenterologists in our study were unanimous in reporting that the discharge policies had increased the complexity of patients seen in the ZSFG gastroenterology clinic. It is possible that the large number of patients previously seen in clinic for follow-up of benign pathology results tended to be simple clinic visits, whereas new patient appointments or visits with patients with chronic GI conditions are more complex. This consideration is relevant generally at the primary care – specialty care interface; if more stable follow-up patients are discharged to primary care, then this may increase the overall complexity of a specialist provider’s patient panel.

Wait times for new patient appointments decreased after the introduction of the discharge criteria. This occurred in the context of other initiatives to improve the primary care-specialty care interface within the San Francisco Health Network (SFHN). For example, an integrated electronic consultation and referral program was introduced in 2005 for gastroenterology, allowing referring clinicians and specialists to communicate electronically to ensure appropriate triage and pre-consultation workup [12] for patients requiring gastroenterology. And in 2012, an intervention was developed to improve the quality of gastroenterology consultation notes [14]. These interventions may have also contributed to improved access to SFHN gastroenterology services, but did not likely have a large impact on wait times for new patient appointments. The electronic consultation and referral program had already reached maturity by 2012 with a stable percentage of patients not scheduled for an ambulatory GI visit. And while improvement in written communication by gastroenterologists is key for care coordination, this intervention did not likely impact wait times for new patients. The adoption of criteria for appropriate discharge to primary care from specialty was thus instrumental in sustainably improving access to specialty care.

Our study has several limitations. First, our survey response rate among PCPs was relatively low at 34%. This may have led to response bias and may limit the generalizability of the survey results. Our study was limited to an electronic survey and did not include provider interviews or focus groups which could have provided richer qualitative information. There may be a limitation in the applicability of the study, as many gastroenterology practices already do not bring patients back to clinic for benign endoscopy and colonoscopy results. Also, as previously mentioned, there were parallel interventions that may have improved GI care coordination in the 2013-2015 time frame, so the discharge criteria may not be solely responsible for the improvement in wait times. Finally, our study did not address the patient experience or patient satisfaction with the discharge process.

## Conclusion

In conclusion, we have shown that PCPs and gastroenterologists are satisfied with an intervention to discharge patients from gastroenterology clinic to primary care after certain endoscopic procedures, despite a slight increase in perceived workload reported by PCPs and a perceived increase in complexity of patients seen in gastroenterology clinic. Study limitations include a low response rate from PCPs. Improving access to specialty care services in the safety net will be crucial as demand for specialty services continues to grow. This model may be applicable to gastroenterology practices or other specialty clinics where access to clinic appointments is a challenge.

## Additional files

Additional file 1: Discharge Criteria.pdf (Title: Post-Endoscopy Discharge Criteria from GI Clinic Back to Primary Care.) (PDF 119 kb)

Additional file 2: PCP Survey.pdf (Survey administered to primary care providers). (PDF 56 kb)

Additional file 3: Gastroenterologist Survey.pdf (Survey administered to gastroenterologists). (PDF 54 kb)

## Abbreviations

EGD: Esophagogastroduodenoscopy
GI: Gastroenterology
PCP: Primary care provider
SFHN: San Francisco Health Network
FOBT: Fecal occult blood test
FIT: Fecal immunochemical test
ZSFG: Zuckerberg San Francisco General Hospital
