# Supplementary material for: Gastroenterologist and primary care perspectives on a post-endoscopy discharge policy: impact on clinic wait times, provider satisfaction and provider workload
Source: BMC Health Serv Res. 2018 Jan 10;18:16. doi: 10.1186/s12913-017-2819-6 (PMC5763538; doi:10.1186/s12913-017-2819-6)
Supplement: Supplementary file 1 — Discharge Criteria.pdf (Title: Post-Endoscopy Discharge Criteria from GI Clinic Back to Primary Care.) (PDF 119 kb) [file 12913_2017_2819_MOESM1_ESM.pdf]

Post-Endoscopy Discharge Criteria from GI Clinic Back to Primary Care

1. Patients who undergo a completed colonoscopy for colorectal cancer screening/surveillance (including positive FOBT/FIT; personal history of colonic polyps or colorectal cancer; or family history of colonic polyps or colorectal cancer), with a good or excellent bowel preparation, who meet the following criteria:
    a. No malignant-appearing lesions are identified, AND
    b. No more than a few small polyps are identified, AND
    c. All identified polyps are completely resected
2. Patients who undergo a completed colonoscopy for hematochezia with a good or excellent bowel preparation who meet the following criteria:
    a. No malignant-appearing lesions or lesions at high risk of bleeding are identified, AND
    b. No more than a few small polyps are identified, AND
    c. All identified polyps are completely resected, AND
    d. Patient has no alarm symptoms or anemia.
3. Patients who undergo a completed upper endoscopy for dyspepsia who meet the following criteria:
    a. No gastric ulcers or malignant-appearing lesions are identified, AND
    b. Patient has no alarm symptoms or anemia
4. Patients who undergo upper endoscopy and colonoscopy for iron deficiency anemia who meet the following criteria:
    a. No cause for iron deficiency anemia identified
    b. No alarm symptoms (e.g. overt GI bleeding, weight loss, fatigue)
