# Supplementary material for: Gastroenterologist and primary care perspectives on a post-endoscopy discharge policy: impact on clinic wait times, provider satisfaction and provider workload
Source: BMC Health Serv Res. 2018 Jan 10;18:16. doi: 10.1186/s12913-017-2819-6 (PMC5763538; doi:10.1186/s12913-017-2819-6)
Supplement: Supplementary file 2 — PCP Survey.pdf (Survey administered to primary care providers). (PDF 56 kb) [file 12913_2017_2819_MOESM2_ESM.pdf]

# Primary Care Survey- Gastroenterology Clinic Discharge

Please complete the survey below.

Thank you!

---

**Safe Discharge to Primary Care: Primary Care Follow-up Survey**

**You are being asked to complete this survey because you are a primary care provider within the San Francisco Health Network who can refer patients for specialty care services at the Zuckerberg San Francisco General Hospital (ZSFG).**

**You may have participated in a survey in 2012-2013 regarding follow-up after referral to gastroenterology clinic. After this survey, new guidelines were developed and implemented for patients meeting certain criteria to be discharged from the ZSFG gastroenterology (GI) clinic directly to primary care (e.g. without a scheduled GI follow-up visit). The purpose of this current survey is to assess how the new guidelines are working and to continue improving coordination of care between referring providers and gastroenterologists.**

| | |
|---|---|
| Did you participate in the survey in 2012-2013 regarding which patients could be discharged back to primary care following endoscopy (without a planned GI follow-up visit)? | ◯ Yes<br>◯ No<br>◯ Not sure |

**Section 1: Comfort with existing discharge criteria**

**For the following clinical scenarios, we would like to know your comfort level with managing the patient in clinic after they have received upper endoscopy or colonoscopy. In these scenarios, the patient is not scheduled for follow-up in clinic with gastroenterology, but formal recommendations are left in the electronic medical record by a gastroenterologist and sent to the patient.**

**History for Clinical Scenario 1: Patient undergoes a colonoscopy for positive FOBT/FIT, personal history of polyps, or family history of polyps/colon cancer. The bowel preparation is good to excellent. Any polyps identified are completed removed.**

**Findings: Normal colonoscopy. Any biopsies taken show normal colonic mucosa.**

**Follow-up: A gastroenterologist reviews the biopsy results and leaves formal recommendations in the electronic medical record. A letter is mailed to the patient regarding the results. The patient is not scheduled for any follow-up visit with gastroenterology after the colonoscopy.**

How comfortable are you caring for this patient in clinic after their colonoscopy?

Not comfortable: You are not comfortable caring for this patient despite receiving formal recommendations from GI.

Very comfortable: You are very comfortable caring for this patient upon receipt of formal recommendations from GI.

○ Not comfortable
○ Mildly uncomfortable
○ Undecided
○ Somewhat comfortable
○ Very comfortable

**History for Clinical Scenario 2: Patient undergoes a colonoscopy for hematochezia. There is no clinical suspicion for an upper GI bleeding source prior to endoscopy. The bowel preparation is good to excellent. Any polyps identified are completely removed.**

**Findings: No cause for hematochezia identified; the patient is not anemic and does not have any other alarm symptoms (e.g.: abdominal pain, weight loss, fatigue). Any biopsies taken are normal.**

**Follow-up: A gastroenterologist reviews any biopsy results and leaves formal recommendations in the electronic medical record. A letter is mailed to the patient regarding the results. The patient is not scheduled for any follow-up visit with gastroenterology after the colonoscopy.**

How comfortable are you caring for this patient in clinic after their colonoscopy?

Not comfortable: You are not comfortable caring for this patient despite receiving formal recommendations from GI.

Very comfortable: You are very comfortable caring for this patient upon receipt of formal recommendations from GI.

- ◯ Not comfortable
- ◯ Mildly uncomfortable
- ◯ Undecided
- ◯ Somewhat comfortable
- ◯ Very comfortable

**History for Clinical Scenario 3: Patient undergoes an EGD for dyspepsia.**

**Findings: Normal EGD. Biopsies are normal, and cause for dyspepsia not identified.**

**Follow-up: A gastroenterologist reviews the biopsy results and leaves formal recommendations in the electronic medical record. A letter is mailed to the patient regarding the results. The patient is not scheduled for any follow-up visit with gastroenterology after the endoscopy.**

How comfortable are you caring for this patient in clinic after their endoscopy?

Not comfortable: You are not comfortable caring for this patient despite receiving formal recommendations from GI.

Very comfortable: You are very comfortable caring for this patient upon receipt of formal recommendations from GI.

○ Not comfortable
○ Mildly uncomfortable
○ Undecided
○ Somewhat comfortable
○ Very comfortable

**History for Clinical Scenario 4: Patient undergoes an EGD and colonoscopy for iron deficiency anemia. The bowel preparation is good to excellent. Any colonic polyps identified are completely removed.**

**Findings: No cause for iron deficiency anemia identified; patient has NO alarm symptoms (e.g. overt GI bleeding, weight loss, fatigue).**

**Follow-up: A gastroenterologist reviews any biopsy results and leaves formal recommendations in the electronic medical record. A letter is mailed to the patient regarding the results. The patient is not scheduled for any follow-up visit with gastroenterology after the colonoscopy/endoscopy.**

How comfortable are you caring for this patient in clinic after their endoscopy?

Not comfortable: You are not comfortable caring for this patient despite receiving formal recommendations from GI.

Very comfortable: You are very comfortable caring for this patient upon receipt of formal recommendations from GI.

- ○ Not comfortable
- ○ Mildly uncomfortable
- ○ Undecided
- ○ Somewhat comfortable
- ○ Very comfortable

## Section 2: Satisfaction with discharge guideline process

**Since 2013, the ZSFG gastroenterology clinic and referring PCPs have implemented discharge criteria in which patients in the previous clinical scenarios can be discharged from gastroenterology clinic to primary care (e.g without a planned GI follow-up visit), with gastroenterologists reviewing the biopsy results, documenting formal recommendations in the electronic medical record and sending a letter to the patient.**

Have you had patients referred for upper endoscopy/colonoscopy who were discharged back to primary care following the procedure, without planned gastroenterology follow-up?

As a reminder, the groups of patients being discharged by this process are:
-Colonoscopy for positive FOBT/FIT, personal history of polyps, or family history of polyps/colon cancer (with normal colonoscopy result)
-Colonoscopy for hematochezia (no cause for hematochezia identified)
-EGD for dyspepsia (normal EGD results)
-EGD/colonoscopy for iron deficiency anemia (no cause for IDA identified)

- ◯ Yes
- ◯ No
- ◯ Not sure

Please rate your degree of satisfaction with the quality of the recommendations provided by gastroenterologists for patients who are discharged to primary care immediately after endoscopy.

- ◯ Very satisfied
- ◯ Satisfied
- ◯ Neither satisfied nor unsatisfied
- ◯ Unsatisfied
- ◯ Very unsatisfied

How satisfied are you with this process for discharging patients from gastroenterology clinic immediately after endoscopy for these specific groups of patients?

- ◯ Very satisfied
- ◯ Satisfied
- ◯ Neither satisfied nor unsatisfied
- ◯ Unsatisfied
- ◯ Very unsatisfied

Section 3: Workload considerations

When patients are discharged back to primary care clinic after their endoscopy, rather than having a scheduled GI follow-up appointment, how does this affect your workload?

- ◯ Lessens workload
- ◯ Slightly lessens workload
- ◯ No effect on workload
- ◯ Slightly increases workload
- ◯ Increases workload

What additional comments do you have about the GI clinic discharge process?

__________________________________________

## Section 4: Background

**Please provide the following demographic information.**

What is your role?

- ○ Physician (attending)
- ○ Physician (resident or fellow)
- ○ Nurse practitioner primarily performing primary care
- ○ Physician assistant primarily performing primary care

What is your year of graduation from medical school or NP/PA training?

__________________________________

What is the location of your clinic?

- ○ San Francisco General Hospital (ZSFG) based primary care
- ○ San Francisco Health Network (SFHN) primary care
- ○ San Francisco Community Clinic Consortium
- ○ Other

How many half-days per week are you directly involved in outpatient care (including personally seeing patients in clinics, precepting trainees in clinic, and performing ambulatory procedures)?

- ○ 0
- ○ 1-2 half-days per week
- ○ 3-4 half-days per week
- ○ 5-6 half-days per week
- ○ At least 7 half-days per week
