# Supplementary material for: Gastroenterologist and primary care perspectives on a post-endoscopy discharge policy: impact on clinic wait times, provider satisfaction and provider workload
Source: BMC Health Serv Res. 2018 Jan 10;18:16. doi: 10.1186/s12913-017-2819-6 (PMC5763538; doi:10.1186/s12913-017-2819-6)
Supplement: Supplementary file 3 — Gastroenterologist Survey.pdf (Survey administered to gastroenterologists). (PDF 54 kb) [file 12913_2017_2819_MOESM3_ESM.pdf]

Gastroenterologist survey

Participant ID ______________________________

Safe Discharge to Primary Care: Gastroenterologist Follow-up Survey

This survey is to evaluate the process for discharging patients from the GI clinic to primary care providers following endoscopy for patients who meet certain discharge criteria. These criteria were developed and implemented through a series of surveys with SFGH gastroenterologists and referring primary care physicians in 2012-2013. The purpose of this survey is to evaluate how the discharge criteria are working.

Section 1: Comfort with existing discharge criteria
For the following clinical scenarios, we would like to know your comfort level with discharging the patient from the gastroenterology clinic after their upper endoscopy or colonoscopy. In these scenarios, the patient is not scheduled for follow-up in gastroenterology clinic , but formal recommendations are left in the medical record for the PCP by a gastroenterologist.

History for Clinical Scenario 1: Patient undergoes a colonoscopy for positive FOBT/FIT, personal history of polyps, or family history of polyps/colon cancer. The bowel preparation is good to excellent. Any polyps identified are completed removed.

Findings: Normal colonoscopy. Any biopsies taken show normal colonic mucosa.

How comfortable are you discharging this patient from gastroenterology clinic after their colonoscopy (without scheduled gastroenterology follow-up)? Not comfortable: You are not comfortable discharging this patient to primary care following endoscopy despite providing formal recommendations from GI documented in the electronic medical record. Very comfortable: You are very comfortable discharging this patient to primary care following endoscopy with formal recommendations from GI documented in the electronic medical record.

- ○ Not comfortable
- ○ Mildly uncomfortable
- ○ Undecided
- ○ Somewhat comfortable
- ○ Very comfortable

How frequently do you discharge patients in this scenario to primary care after colonoscopy (as opposed to scheduling a follow-up gastroenterology visit)?

- ○ Never
- ○ Sometimes
- ○ Most of the time
- ○ Almost always
- ○ Always

History for Clinical Scenario 2: Patient undergoes a colonoscopy for hematochezia. There is no clinical suspicion for an upper GI bleeding source prior to endoscopy. The bowel preparation is good to excellent. Any polyps identified are completely removed.

Findings: No cause for hematochezia identified; the patient is not anemic and does not have any other alarm symptoms (e.g.: abdominal pain, weight loss, fatigue). Any biopsies taken are normal.

How comfortable are you discharging this patient from gastroenterology clinic after their colonoscopy (without scheduled gastroenterology follow-up)? Not comfortable: You are not comfortable discharging this patient to primary care following endoscopy despite providing formal recommendations from GI documented in the electronic medical record. Very comfortable: You are very comfortable discharging this patient to primary care following endoscopy with formal recommendations from GI documented in the electronic medical record.

- ◯ Not comfortable
- ◯ Mildly uncomfortable
- ◯ Undecided
- ◯ Somewhat comfortable
- ◯ Very comfortable

How frequently do you discharge patients in this scenario to primary care after colonoscopy (as opposed to scheduling a follow-up gastroenterology visit)?

- ◯ Never
- ◯ Sometimes
- ◯ Most of the time
- ◯ Almost always
- ◯ Always

History for Clinical Scenario 3: Patient undergoes an EGD for dyspepsia.

Findings: Normal EGD. Biopsies are normal, and cause for dyspepsia not identified.

How comfortable are you discharging this patient from gastroenterology clinic after their endoscopy (without scheduled gastroenterology follow-up)? Not comfortable: You are not comfortable discharging this patient to primary care following endoscopy despite providing formal recommendations from GI documented in the electronic medical record. Very comfortable: You are very comfortable discharging this patient to primary care following endoscopy with formal recommendations from GI documented in the electronic medical record.

- ◯ Not comfortable
- ◯ Mildly uncomfortable
- ◯ Undecided
- ◯ Somewhat comfortable
- ◯ Very comfortable

How frequently do you discharge patients in this scenario to primary care after endoscopy (as opposed to scheduling a follow-up gastroenterology visit)?

- ◯ Never
- ◯ Sometimes
- ◯ Most of the time
- ◯ Almost always
- ◯ Always

History for Clinical Scenario 4: Patient undergoes an EGD and colonoscopy for iron deficiency anemia. The bowel preparation is good to excellent. Any colonic polyps identified are completely removed.

Findings: No cause for iron deficiency anemia identified; patient has NO alarm symptoms (e.g. overt GI bleeding, weight loss, fatigue).

How comfortable are you discharging this patient from gastroenterology clinic after their colonoscopy/endoscopy (without scheduled gastroenterology follow-up)? Not comfortable: You are not comfortable discharging this patient to primary care following endoscopy despite providing formal recommendations from GI documented in the electronic medical record. Very comfortable: You are very comfortable discharging this patient to primary care following endoscopy with formal recommendations from GI documented in the electronic medical record.

- ◯ Not comfortable
- ◯ Mildly uncomfortable
- ◯ Undecided
- ◯ Somewhat comfortable
- ◯ Very comfortable

How frequently do you discharge patients in this scenario to primary care after colonoscopy/endoscopy (as opposed to scheduling a follow-up gastroenterology visit)?

- ◯ Never
- ◯ Sometimes
- ◯ Most of the time
- ◯ Almost always
- ◯ Always

Section 2: Satisfaction with the new discharge process

Since 2013, the SFGH gastroenterology clinic and referring PCPs have implemented discharge criteria in which patients in the previous clinical scenarios can be discharged from gastroenterology clinic to primary care (e.g. without a planned GI follow-up visit), with gastroenterologists reviewing the biopsy results, documenting formal recommendations in the electronic medical record and sending a letter to the patient.

How satisfied are you in general with the process for discharging patients from gastroenterology clinic immediately after endoscopy for patients who meet these criteria?

- ◯ Very satisfied
- ◯ Satisfied
- ◯ Neither satisfied nor unsatisfied
- ◯ Unsatisfied
- ◯ Very unsatisfied

Section 3: Workload considerations

For patients to be discharged from gastroenterology clinic after endoscopy via this process, how does discharging the patient to primary care with anticipatory guidance affect your workload (compared with seeing the patient for a follow-up visit)?

- ◯ Lessens workload
- ◯ Slightly lessens workload
- ◯ No effect on workload
- ◯ Slightly increases workload
- ◯ Increases workload

Comments (optional): ______________________________

Have you noticed a change in the average complexity of your clinic patients since the implementation of these discharge criteria?

- ◯ More complex
- ◯ Slightly more complex
- ◯ No change
- ◯ Slightly less complex
- ◯ Less complex

Comments (optional): ______________________________

If you have suggestions for modifying or improving the clinic discharge process, please describe here: ______________________________

Background

How many half-days per week do you spend performing endoscopic procedures?

- ◯ 0
- ◯ 1-2 half-days per week
- ◯ 3-4 half-days per week
- ◯ 5-6 half-days per week
- ◯ At least 7 half-days per week

How many half-days per week do you spend in ambulatory clinic, either providing patient care directly or overseeing fellows/residents?

- ◯ 0
- ◯ 1-2 half-days per week
- ◯ 3-4 half-days per week
- ◯ 5-6 half-days per week
- ◯ At least 7 half-days per week
